# The American Society of Dental Surgeons

**Published:** 1854-01

**Authors:** 


### THE AMERICAN SOCIETY OF DENTAL SURGEONS.

This time-honored association, at its last meeting, revised and amended its constitution, and passed several important by-laws. A committee consisting of Drs. J. D. White and . E. Townsend, were appointed to print the revised constitution and by-laws. We have not yet met with this in print, but we understand that the fee for admission is increased to $25, and we see the following by-law reported : Resolved, "That any member of this Society, who shall extol his own peculiar merits over a fellow practitioner in the public prints, or employ means of advertisement which may be regarded by this Society as lowering its dignity or compromising its character, shall be impeached, suspended or expelled." This is very similar in purport and phraseology to a resolution of the Mississippi Association, adopted at its second annual meeting. We see, also the following, reported as the XIII Article of the Constitution : "Dental Ethics —Any dentist, who shall procure a patent for a remedy or instrument of dental surgery, or who deals in patent remedies or nostrums, shall be disqualified for membership." We see by an after decision of the Society, that its members are allowed to use patented instruments. A distinction then exists between a "remedy or nostrum," and an "instrument." It is, therefore, the operation and not the instrument, that is the remedy for the disease.

We cannot but express the regret that our venerated President, Dr. E. Parmly should have felt constrained to decline the office any longer. We feel assured that the Association partake in this regret. We hope he may still meet with the Society, and that we shall have the pleasure to welcome him in the Queen City at the next meeting. The Society, in its present presiding officer, Dr. E. Townsend, will have a dignified, courteous and able President.

